# Endogenous pararetroviral sequences in tomato (*Solanum lycopersicum*) and related species

**DOI:** 10.1186/1471-2229-7-24

**Published:** 2007-05-21

**Authors:** Christina Staginnus, Wolfgang Gregor, M Florian Mette, Chee How Teo, Eduviges Glenda Borroto-Fernández, Margit Laimer da Câmara Machado, Marjori Matzke, Trude Schwarzacher

**Affiliations:** 1 Gregor Mendel Institute of Plant Molecular Biology (GMI), 1030 Wien, Austria; 2 Research Institute of Biochemical Pharmacology and Molecular Toxicology, University of Veterinary Medicine, 1210 Wien, Austria; 3 AG Epigenetik, Institute of Plant Genetics and Crop Plant Research (IPK) Gatersleben, 06466 Gatersleben, Germany; 4 Department of Biology, University of Leicester, Leicester LE1 7RH, UK; 5 Institute of Applied Microbiology, University of Natural Resources and Applied Life Sciences (BOKU), 1190 Wien, Austria

## Abstract

**Background:**

Endogenous pararetroviral sequences (EPRVs) are a recently discovered class of repetitive sequences that is broadly distributed in the plant kingdom. The potential contribution of EPRVs to plant pathogenicity or, conversely, to virus resistance is just beginning to be explored. Some members of the family Solanaceae are particularly rich in EPRVs. In previous work, EPRVs have been characterized molecularly in various species of *Nicotiana *including *N.tabacum *(tobacco) and *Solanum tuberosum *(potato). Here we describe a family of EPRVs in cultivated tomato (*Solanum lycopersicum *L.) and a wild relative (*S.habrochaites*).

**Results:**

Molecular cloning and DNA sequence analysis revealed that tomato EPRVs (named *Lyc*EPRVs) are most closely related to those in tobacco. The sequence similarity of *Lyc*EPRVs in *S.lycopersicum *and *S.habrochaites *indicates they are potentially derived from the same pararetrovirus. DNA blot analysis revealed a similar genomic organization in the two species, but also some independent excision or insertion events after species separation, or flanking sequence divergence. *Lyc*EPRVs share with the tobacco elements a disrupted genomic structure and frequent association with retrotransposons. Fluorescence in situ hybridization revealed that copies of *Lyc*EPRV are dispersed on all chromosomes in predominantly heterochromatic regions. Methylation of *Lyc*EPRVs was detected in CHG and asymmetric CHH nucleotide groups. Although normally quiescent EPRVs can be reactivated and produce symptoms of infection in some *Nicotiana *interspecific hybrids, a similar pathogenicity of *Lyc*EPRVs could not be demonstrated in *Solanum *L. section *Lycopersicon *[Mill.] hybrids. Even in healthy plants, however, transcripts derived from multiple *Lyc*EPRV loci and short RNAs complementary to *Lyc*EPRVs were detected and were elevated upon infection with heterologous viruses encoding suppressors of PTGS.

**Conclusion:**

The analysis of *Lyc*EPRVs provides further evidence for the extensive invasion of pararetroviral sequences into the genomes of solanaceous plants. The detection of asymmetric CHH methylation and short RNAs, which are hallmarks of RNAi in plants, suggests that *Lyc*EPRVs are controlled by an RNA-mediated silencing mechanism.

## Background

Plant pararetroviruses (*Caulimoviridae*) have double-stranded DNA genomes and are considered retroelements because they use reverse transcription for replication. Unlike other retroelements, such as retroviruses and retrotransposons, integration into the host genome is not essential during their replication cycle. Nevertheless, in recent years there have been accumulating reports of endogenous pararetroviral sequences (EPRVs) in the nuclear genomes of several plants including tobacco (*Nicotiana tabacum*) and other *Nicotiana *species [1-3], potato [4], banana [5-7], petunia [8] and rice [9]. EPRVs are assumed to integrate by illegitimate recombination into the host genome, where they may accumulate to high copy numbers [1,10]. Although EPRVs are being detected in an increasing number of plant species, the detailed structure of individual EPRV integrants and flanking regions has been analysed only in a few families [1,3,6,8,9].

The role of EPRVs in plant-virus interactions is not yet fully understood. Current information suggests that EPRVs are not always neutral components of plant genomes but can potentially contribute to either pathogenicity or virus resistance in the host. Indeed, integrated sequences of *Banana streak virus *(BSV), *Tobacco vein clearing virus *(TVCV) and *Petunia vein clearing virus *(PVCV) [2,5,6,8] can be reactivated in response to abiotic or genomic stress. Episomal copies are probably formed by transcription from tandemly arranged integrants or recombination from fragmented integrants [6,8], which leads to the assembly of virus particles and symptoms of virus infection. Interspecific crosses and *in vitro *propagation can induce EPRV reactivation, which has been shown to be economically detrimental in banana breeding [2,6,11-13].

Under different conditions or in other genome constitutions, EPRVs remain silent and might even have beneficial effects for their hosts by providing virus resistance via homology-dependent transcriptional or posttranscriptional gene silencing [1,14]. Consistent with this proposal, EPRV-derived enhancer-promotor sequences integrated as transgenes into tobacco chromosomes became silenced and methylated in the presence of homologous EPRVs [15]. Homology-dependent silencing can be induced by several interrelated pathways [16] that involve aberrant or double stranded RNA that is processed to short RNAs by RNaseIII-like enzymes (Dicer). Post-transcriptional gene silencing (PTGS), which is the plant equivalent of RNAi, is able to counteract RNA and DNA viruses at the mRNA level [17,18]. In addition, RNA-mediated epigenetic modifications, such as RNA-directed DNA or histone methylation [19], could transcriptionally repress DNA viruses at the chromatin level. Further elucidation of host control over EPRVs will not only facilitate assessment and the prevention of EPRV reactivation but may also suggest strategies for genetically engineering pathogen resistance in agriculturally important plants.

Studies so far indicate that EPRVs are abundant in some members of the family Solanaceae, an economically important taxon that includes tobacco, petunia, potato, (bell) pepper (*Capsicum annuum*) and tomato. In addition to its role as an important food crop, cultivated tomato, *S.lycopersicum *subsection lycopersicon, represents a model plant within this family with a small diploid genome that lacks large duplications (2n = 24, size 953 Mb; [20]), with a high-density genetic map [21], and large mutant collection . Recently, it has been chosen for sequencing by an international consortium [22]. Repetitive sequences comprise wide blocks of pericentromeric heterochromatin in the tomato genome [23,24] that nevertheless also harbour a considerable share of genic sequences [25,26]. In an *S.lycopersicum *(Heinz 1706) BAC library [24], 194 of the 1205 sequenced-tagged connectors (STCs) were similar to retrotransposons and four were similar to tobacco EPRVs, although these sequences were not characterized further.

To increase our understanding of endogenous pararetroviral sequences in economically relevant, genetically tractable crops, we have characterized a family of EPRVs in *S.lycopersicum *and a wild relative, *S.habrochaites *which is exploited in crosses with *S.lycopersicum *to introgress favourable traits [27,28] with respect to sequence and structure of a number of integrated copies, as well as to chromosomal localization. In addition, we have analysed the methylation status of the EPRV integrants and their transcriptional activity in *S.lycopersicum*, *S.habrochaites *and interspecific hybrids to investigate the nature of host control of these sequences.

## Results

### LycEPRV identification, isolation and sequence analysis

**Figure 1:**
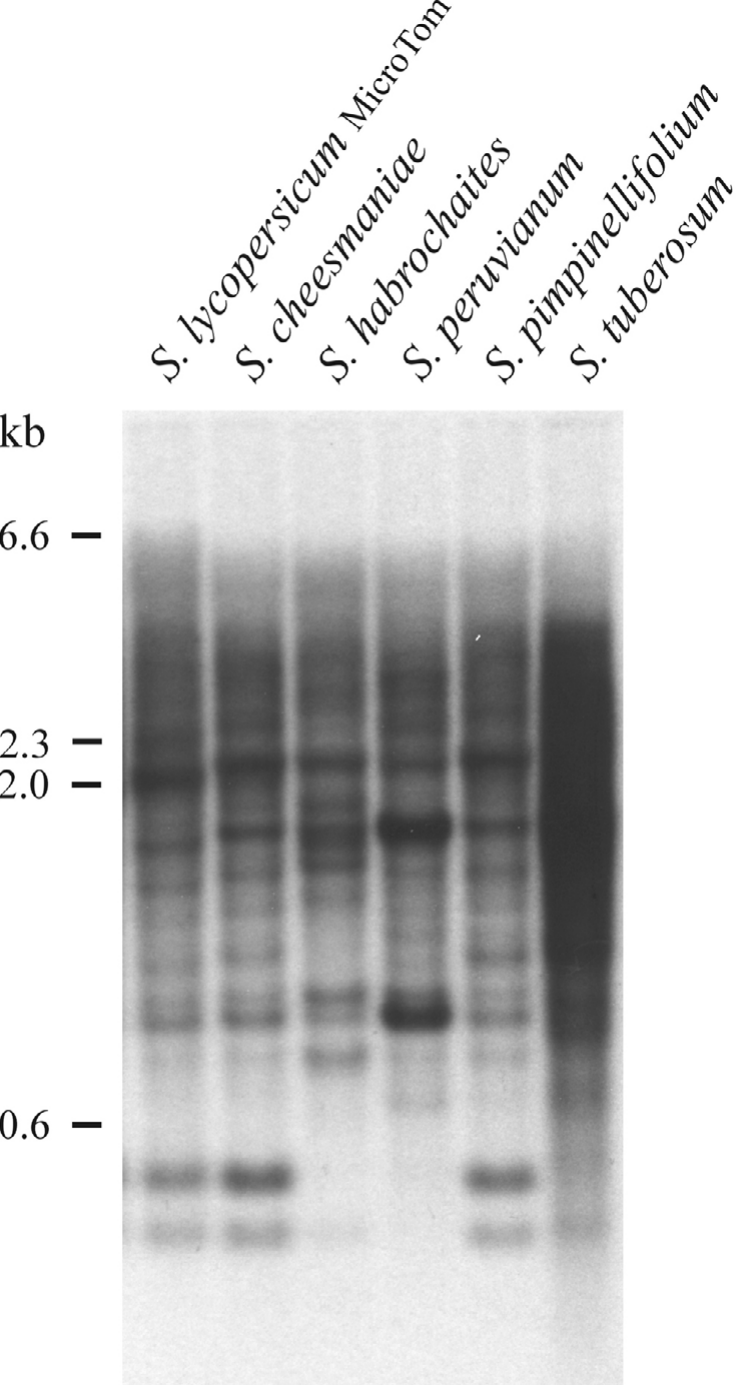
**Genomic organization of EPRV sequences in the genus *Solanum *subsection *Lycopersicon***. DNA preparations from five species of the genus *Solanum *subsection *Lycopersicon *and *Solanum tuberosum *were restricted with *Xba*I and hybridized to a 5.5 fragment of *Ns*EPRV covering ORF 2 to 4 and the IGR. Similar data (lanes 1 to 5) have been shown previously [47].

Tomato EPRVs were originally detected by DNA blot analysis using a 5.5 kb DNA fragment of *Ns*EPRV (*Nicotiana sylvestris *EPRV), one of three EPRV families in tobacco [1,2], to probe DNA prepared from various species of *Solanum*. The resulting banding pattern was complex, with numerous strong and weak bands superimposed on a background smear (Fig.1). This pattern is reminiscent of that observed with *Nicotiana *species [1] and suggests a dispersed organization of multiple copies of a related EPRV family. Judging from the hybridization intensity, the relative copy number of the elements detected by the *Ns*EPRV probe was similar in all five *Solanum *species tested. The banding pattern in *S.lycopersicum *strongly resembled that in *S.cheesmaniae *and *S.pimpinellifolium*, whereas notable differences were observed in *S.habrochaites *and *S.peruvianum *(Fig.1).

**Figure 2:**
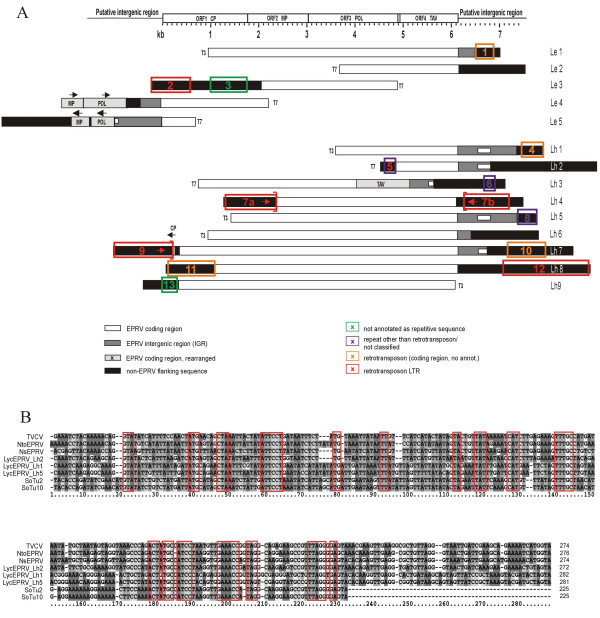
**A. Analysis of cloned *Lyc*EPRV sequences and flanking sequences**. Alignment of cloned EPRV sequences from *S.lycopersicum *(Le1-5) and *S.habrochaites *(Lh1-9) to the structure of TVCV-like EPRVs comprising four ORFs (upper bar): coat protein (CP), cell-to-cell movement protein (MP), polyprotein (POL) and transactivator domain (TAV). Rearranged coding regions are indicated by extra boxes and arrows for a deviating orientation of the reading frames. Nine clones contain parts of the intergenic region (IGR) marked by grey boxes with a white square for the position of the conserved 272 to 282 bp-box. Black bars indicate flanking sequences unrelated to EPRVs. Survey of sequences flanking the EPRVs in *S.lycopersicum *and *S.habrochaites *is given by coloured boxes. The majority represents repetitve elements (orange, red and blue boxes) most of which belong to retrotransposons (orange and red boxes), especially the LTR regions (red boxes). Arrows point towards the end of similar LTRs which is marked by a bracket. A description of the flanking sequences is listed in Table 1 according to the numbers. **B. Sequence conservation within a 272to 282 bp box of the IGR from differentSolanaceae EPRVs**. Alignment of the respective region of three *Lyc*EPRVs (Lh1, Lh2, Lh5) to three different tobacco EPRVs (TVCV, [2]; *Ns*EPRV, [1]; *Nto*EPRV, [3]) and to two *Solanum tuberosum *EPRV copies (SoTuI-2, SoTuI-10; AJ564214, AJ564220; [4]). Next to a remarkable overall sequence homogeneity within the IGR region several shorter motives are highly conserved between EPRVs from all three species (red frames).

To analyze the tomato EPRV sequences in more detail, a genomic λ-library was constructed from cultivated tomato (*S.lycopersicum *"MicroTom"; [29]) and the wild relative *S.habrochaites*. Both λ-libraries were screened with the 5.5 kb fragment of *Ns*EPRV. Five positive clones were isolated and partly sequenced for *S.lycopersicum *and nine for *S.habrochaites*. Each clone contained EPRV-like DNA and flanking plant genomic sequences (Fig.2A, Table 1).

**Table 1:** Description of identified sequences flanking the *Lyc *EPRVs. Numbers correspond to those shown in Fig.2A.

**No.**	**Clone**	**Description**	**Position in clone**
1.	Le1	Integrase ORF, *Solanum demissum *retrotransposon (AAT39954);	5431–5846
		polyprotein ORF, *S.lycopersicum *retrotransposon (AAD13304); PCRT2-2, *S.lycopersicum *pericentromeric retrotransposon (AY850394);	5800–5968,
		PCRT1c-1 S. lycopersicum pericentromeric retrotransposon (AY850394).	5477–5498
2.	Le3	Putative solo-LTR, UTRT3, tandem repeat (AC139840; AY850394).	1–797
3.	Le3	Putative DEAD/DEAH box, RNA helicase protein ORF, *A. thaliana *(AAO00880), *O.sativa *(AAN62787).	1237–1992
4.	Lh1	putative coding region, PCRT1b-3 *S.lycopersicum *pericentromeric retrotransposon (AY850394);	3879–4211,
		putative coding region, Caterina-2 S*.lycopersicum Ty3-gypsy *retrotransposon (AY678298).	3901–4212
5.	Lh2	putative LTR, PCR1b *S.lycopersicum *pericentromeric retrotransposon (AY850394).	98–332
6.	Lh3	TNP2 like transposon protein (Tpase), class II transposon, *O.sativa *(AAM18727).	5925–5990
7.(a, b)	Lh4	LTR ends, inverted, PCRT1a *S.lycopersicum *pericentromeric retrotransposon (AY850394).	131–1076; 4868–6132
8.	Lh5	51bp repeat motif;	5870–6206
		unclassified transposable element XC (AY678298);	6156–6171
		unclassified transposable element XB (AY678298).	6218–6252
9.	Lh7	LTR end, PCRT1a (as in Lh4) S*.lycopersicum *pericentromeric retrotransposon (AY850394).	11–1257
10.	Lh7	not annotated pericentromeric repeat; PCRT1b S*.lycopersicum *pericentromeric retrotransposon (AY850394).	8132–8988
11.	Lh8	PCTR1a *S.lycopersicum *pericentromeric retrotransposon (AY850394); TGRII dispersed repetitive sequence (AY880062).	49–1000
12.	Lh8	Solo-LTR, PCRT1d & PCRT1g *S.lycopersicum *pericentromeric retrotransposon (AY850394); not annotated sequences from pericentric heterochromatin;	6863–8656
		root knot nematode resistence marker (DQ090954).	8108–8320
13.	Lh9	root knot nematode resistence marker (DQ090954); *S.habrochaites *RGA marker sequence (AF534327).	414–716

EPRV-like sequences from both species were AT-rich (65.4–78.4%) and were most similar to EPRVs in *Nicotiana*, revealing up to 83% sequence identity to endogenous *Tobacco vein clearing virus *(TVCV; [2]), *Ns*EPRV [1], and *Nto*EPRV (*N. tomentosiformis *EPRV; the second EPRV family in tobacco; [3]). Similar to the *Nicotiana *EPRVs, four open reading frames (ORFs) were identified (Fig.2A): coat protein (CP), cell-to-cell movement protein (MP), polyprotein (POL) and transactivator protein (TAV). The POL domain revealed 80 to 90% identical nucleotides, compared to MP (75 to 91%) and TAV (63 to 95%). Only one clone contained a full CP sequence that showed 65 to 94% sequence identity to fragments of CP sequences from other clones. The identity between DNA sequences derived from the same species (*S.lycopersicum *or *S.habrochaites*) was generally not higher than between species. Thus, in the subset of clones analyzed, no species-specific clusters of identity were identified and sequences within one species are as divergent as between species. We therefore assigned these sequences to a single family termed *Lyc*EPRV (*Lycopersicon *endogenous pararetrovirus).

The putative amino acid sequence identities of the coding regions ranged from 60 to 87% identity for MP, 72 to 89% for POL and 48 to 91% for TAV (CP shares 39 to 85% identity to various fragments). However, all of the cloned protein-coding regions are either truncated or harbour several frameshifts and stop codons and can therefore be considered translationally defective, a feature also found with *Nicotiana *EPRVs. Nine of the clones contained parts of the putative non-coding intergenic region (IGR) of the virus. The IGR was less conserved compared to the ORFs except for a 272 to 282 bp box (Fig.2B) which revealed up to 86 to 92% sequence identity on the nucleotide level. The conserved 272 to 282 bp box has an overall identity of up to 70% with its counterpart in *S.tuberosum*, SoTu [4] and 80% to the IGR of *Nicotiana *EPRVs with several highly conserved motives. Some IGR sequences contained short (27 to 104 bp) AT-rich structures of low complexity (Lh2, Lh5, Le4, Le5) while others revealed short (12 to 24 bp) direct repeats which were not conserved between the different IGRs (Lh2, Lh5, Lh7, Le5). Some clones (Lh7, Lh2, Lh3) contain a conserved 12 bp motif complementary to the 3'end of the tRNA^Met ^(5'-TGGTATCAGAT/GC-3') 50 to 60 bp upstream of this box as well as a putative polyadenylation signal (5'-AATAAA-3') and a putative TATA box (5'-TATAAA-3') at a distance of 130 to 140 bp and 150 to 160 bp upstream, respectively.

All of the cloned *Lyc*EPRV sequences were truncated and flanked either by plant DNA unrelated to EPRVs or by rearranged (fragmented, inverted or otherwise partly duplicated) EPRV regions that appeared to be out of context when compared to the TVCV-like consensus structure (Le4, Le5, Lh3, Lh7; Fig.2A). Nearly all *Lyc*EPRV junctions analysed adjoin transposable elements, most frequently retrotransposon LTRs or related sequences (see Table 1 and Fig.2A). Clones from *S.habrochaites *revealed homologies to members of the PCRT1 family, a *Ty3-gypsy *(*Metaviridae*) element that is dispersed throughout the pericentric heterochromatin of *S.lycopersicum *(AY850394; [30]). The LTRs of PCRT1 partly correspond to the repetitive families TGRII and U30, the latter of which comprises more than 4000 copies in the *S.lycopersicum *genome [30,31]. The junctions between EPRV and PCRT1 sequences were verified for three clones by PCR amplification from genomic DNA (Lh2, Lh4 and Lh7, data not shown), confirming that the *Lyc*EPRV sequences are indeed physically joined to plant DNA while these sequences could not be amplyfied in *S.lycopersicum*.

We reconstructed a general structure from the alignments of several incomplete sequences (upper bar in Fig.2A). The coding region closely resembles that of the tobacco elements (*Ns*EPRV, *Nto*EPRV) in size with 1779 bp for CP, 1293 bp for MP, 1933 bp for POL which overlaps with TAV (1279 bp) forming a coding region of 6221 bp. The intergenic region varies between 1606 to 1680 bp for different clones, summing up to a total length of approx. 7900 bp (7827 to 7901 bp) for a putative full copy of *Lyc*EPRV. The 140 kb sequence of a BAC clone (AC171732) that was submitted only recently (November 2006, note added in revision) revealed a single *Lyc*EPRV copy. A single stretch of 6125 bp of this sequence corresponds to the putative *Lyc*EPRV coding region and reveals the same order of the four ORFs as reconstructed from the λ-clones. The coding region is flanked by altogether 1542 bp homologous to the IGR on both sides and reveals only one internal stop codon. The nucleotide sequence of this copy contains 84–96% identical nucleotides compared with the λ-clones and 76–92% homology to TVCV. Approximately 2.7 kb upstream of this LycEPRV copy sequences homologous to the LTR of PCRT1a could be identified.

### Fluorescent *in situ *hybridization (FISH)

**Figure 3:**
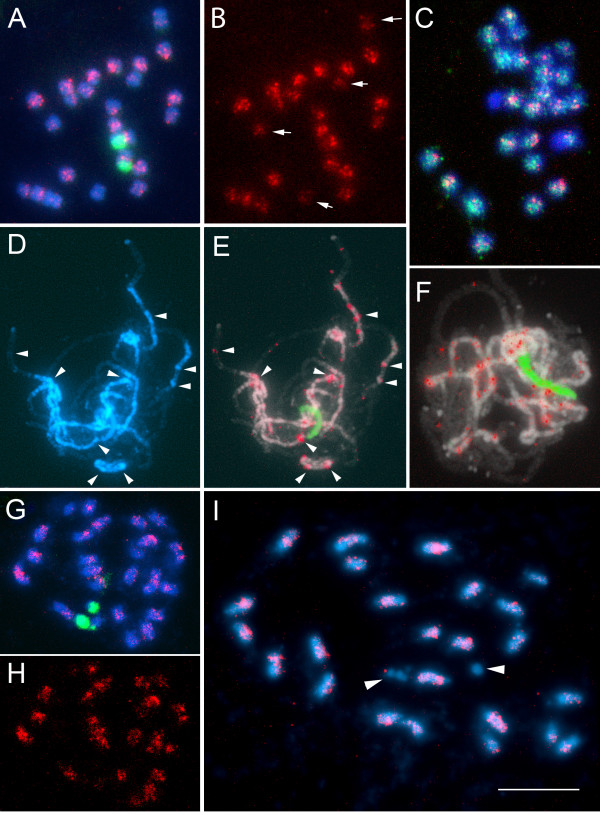
**Chromosomal localization of *Lyc*EPRVs**. Double target fluorescent *in situ *hybridization was carried out on root tip metaphases and male meiotic pachytene cells of *S.lycopersicum *(**A-F**) and S.*habrochaites *(**G-I**). Biotin labelled pooled probes of *Lyc*EPRVs from S.*lycopersicum *(*Lyc*EPRV-Sl, A-F) and *S.habrochaites *(*Lyc*EPRV-Sh, G-I), respectively, that cover most of *Lyc*EPRV sequence (for clone combinations see Table 2) were detected by red Alexa-594 fluorescence and hybridized together with digoxigenin labelled repeated DNA probes detected by green FITC fluorescence. Chromosomes were counterstained with DAPI (blue fluorescence). **A-C**) Metaphase chromosomes of *S.lycopersicum *(2n = 24). *Lyc*EPRV-Sl sequences (red in B and magenta in the overlay with blue DAPI staining in A) are located at the centromeres of most chromosomes with variable intensity, but are absent from the NOR region (green rDNA probe in A) and reduced on four chromosomes (arrows in B). In C the *Lyc*EPRVs are shown to co-localize with the retroelement sequence U30 from *S.lycopersicum *(green) that shows dispersed signals on all chromosomes. **D-F**) Pachytene chromosomes of *S.lycopersicum *are much more extended than metaphase chromosomes and show differentiation with DAPI into strongly stained heterochromatin and weakly stained euchromatin (D). The red *Lyc*EPRV signal is almost exclusively seen in the pericentromeric heterochromatic regions and intercalary chromocentre (arrowheads in D and E), but not at the NOR region (green in E, F; DAPI is shown as grey image with the probe signal falsely coloured red and green, respectively). **G-I**) Metaphase chromosomes of *S.habrochaites *(2n = 24). *Lyc*EPRV-Sh sequences (red in H, magenta in the overlay with blue DAPI staining in G, I) are located near the centromeres of most chromosomes showing stronger signal in some. No signal is visible in the NOR regions (green rDNA probe in G, arrow heads in I). Bar 10 μm.

To analyze the chromosomal distribution of *Lyc*EPRVs, we performed FISH on root tip metaphase chromosomes and pollen mother cells at meiotic prophase of *S.lycopersicum *and *S.habrochaites*. By mixing several probes covering most of the *Lyc*EPRV (*Lyc*EPRV-Sl; Table 2), we were able to observe several weak *Lyc*EPRV-Sl hybridization sites with signal strength of several magnitudes lower than that observed with the control 45S rDNA probe. Sites were visible in varying number near the centromeres of most *S.lycopersicum *chromosomes (Fig.3A, B): there were four to six chromosomes with a stronger signal, four chromosomes showing very weak signals (arrows) and no signal in the NOR region. Similar results were obtained with extended pachytene chromosomes demonstrating that the EPRV signals were located mainly in the DAPI positive pericentromeric heterochromatin or intercalary chromocentres (Fig.3D, E arrowheads), but rarly in the euchromatin. The weak, but in cases distinct signals of varying size and arrangements indicate that probably only few copies of *Lyc*EPRV-Sl are integrated in each cluster, that they might not contain all parts of the probe used or that sequences are only partly conserved. The FISH data (Fig.3A–C) support the results from Southern hybridization (Fig.1) and cloning as well as sequencing data derived from λ-clones (Fig.2) and the BAC clone AC171732 indicating that LycEPRV-Sl are probably not arranged in perfect tandem arrays, are truncated and frequently degenerated.

**Table 2:** Origin of fragments mixed for pooled FISH probes (*Lyc *EPRV-Sl and *Lyc *EPRV-Sh) covering most of the EPRV.

**Pooled probe**	**Derived from region**	**Derived from λ-clone**	**Length**
**LycEPRV-Sl**	CP/MP	Le4 (position: 2882 – 4182)	1300 bp
	MP	Le3 (position: 2365 – 3185)	820 bp
	TAV	Le2 (position: 1260 – 2365)	1100 bp
	IGR (box)	Le5 (position: 2203 – 3207)	1000 bp
**LycEPRV-Sh**	CP	Lh7 (position: 1576 – 2455)	880 bp
	MP	Lh6 (position: 1186 – 1862)	680 bp
	TAV	Lh4 (position: 3617 – 4706)	1090 bp
	IGR (box)	Lh7 (position: 7133 – 7716)	600 bp

FISH of *Lyc*EPRV-Sl in combination with the retroelement sequence U30 on metaphases (Fig.3C) and pachytene chromosomes (see Additional file 1) showed signal from both sequences near the centromeres. The signal of the U30 probe covered a larger area of the centromeric heterochromatin while the *Lyc*EPRV-Sl hybridization signal appeared to be nested within the U30 hybridizing regions. The U30 signal, as the *Lyc*EPRV-Sl signal, was absent from the NOR regions (Fig.3C) as has been previously reported [32]. FISH of *Lyc*EPRV-Sh (Table 2) on metaphase chromosomes of *S.habrochaites *showed similar, but not identical hybridization patterns to *Lyc*EPRV-Sl on *S.lycopersicum *in the pericentromeric region of most chromosomes (Fig.3G–I). However, the signal strength seemed to be more variable between chromosomes (Fig.3I); again, there was no hybridization detected to the NOR region (Fig.3G).

### DNA methylation analysis

**Figure 4:**
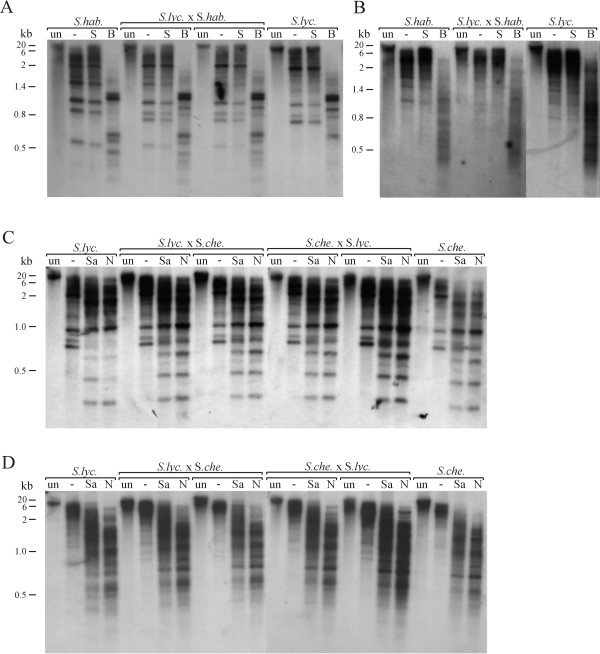
**Analysis of cytosine methylation in *Lyc*EPRV sequences**. DNA of parental plants (flanking) and interspecific hybrids (central) was restricted with *Xba*I (lane 2 to 4 each) and either *Scr*FI (S) and *Bst*NI (B) to detect CHG methylation (A, B) or *Sau*3aI (Sa) and *Nde*I (N) for asymmetric cytosine sites (C, D), the first enzyme of each pair being methylation sensitive. The first lane each contains undigested DNA (un). **A, C. **DNAs were hybridized to a 1.3 kb fragment of the CP/MP reading frame (E1) of a *S.lycopersicum *EPRV copy (Le1) and **B, D. **to a 580 bp fragment of the IGR (H7) of a *S.habrochaites *clone (Lh7).

Cytosine methylation of *Lyc*ERPVs in *S.lycopersicum *and *S.habrochaites *was investigated using methylation-sensitive restriction enzymes and DNA blot analysis. Previous work on EPRVs in *Nicotiana *has shown that the isoschizomer pair *Hpa*II/*Msp*I (recognition sequence CCGG), which is normally used to study CG methylation in animals, is not informative because of frequent CHG methylation in plants, that inhibits both *Hpa*II and *Msp*I, in these sequences [15]. We therefore focused on enzymes sensitive to CHG and CHH methylation: *Scr*FI-*Bst*NI (C^m^CNGG or CCWGG, respectively) reports on CHG methylation while *Sau*3AI-*Nde*I (GAT^m^C) reports on methylation in potentially non-symmetrical cytosines, depending on the sequence context. The first enzyme in each isoschizomer pair is methylation-sensitive. Following a predigestion with *Xba*I, an additional digest was performed with either the methylation-sensitive or -insensitive enzyme from a particular isochizomer pair. Southern blots of electrophoretically separated DNA were hybridized to two different probes each (Fig.4). One was the 1.3 kb fragment (probe E1) of the CP/MP reading frame of a cloned *S.lycopersicum *EPRV copy (Le1), the other one was derived from a *S.habrochaites *clone (Lh7) and comprises 580 bp of the IGR including most of the 273 bp box (probe H7).

For both species, the methylation-sensitive *Scr*FI cleaved little beyond the *Xba*I predigest whereas methylation-insensitive *Bst*NI digested substantially more, indicating the presence of CHG methylation of *Lyc*EPRV sequences (Fig. 4A, B). Little difference between coding regions and IGRs was observed. Hybridization of both the *Sau*3AI-and *Nde*I digested DNA with the CP/MP probe (E1) revealed substantial cleavage compared to the *Xba*I predigestion, suggesting little asymmetrical CHH methylation within the coding EPRV sequences (Fig.4C). Reprobing of the same blot with the IGR probe (H7) revealed a similar pattern, although smaller bands in the *Nde*I digests were more emphasized (Fig.4D). This suggests that asymmetrical methylation of the intergenic region is low but slightly stronger than in coding regions. The sequence of the cloned *Lyc*EPRV sequences did not reveal striking differences in the relative number of CHG and CHH residues between IGR and coding regions.

### Expression analysis

Even though the *Lyc*EPRVs sequenced are defective and unable to encode intact viral proteins, one or more full-length copies could exist and potentially be pathogenic if activated under stress conditions. To test this possibility, we made inter-specific crosses with the aim of provoking a genome stress and then examined the hybrids for symptoms of virus infection. Four different interspecific crosses were made between different wild species (*S.pimpinellifolium*, *S.habrochaites*, *S.cheesmaniae *and, *S.peruvianum*) and *S.lycopersicum *("MicroTom"). The phenotype of 7–27 individuals per cross resembled the phenotype of the wild parent rather than the dwarf cultivar of *S.lycopersicum *("MicroTom"). Their hybrid nature was confirmed by SSR marker analysis (LE 20592; [33]) to exclude selfed offspring.

No typical symptoms of virus-induced diseases could be detected at any time during the development of the hybrids that were grown in a greenhouse for a full year and trimmed frequently. In addition, hybridization of undigested, genomic DNA of selected individuals to probe E1 and H7 (coding region and IGR, respectively) failed to demonstrate episomal virus DNA since all individuals lacked the expected three bands for the linear, circular or supercoiled episomal DNA species (Fig.4, first lane each).

The cytosine methylation of the interspecific hybrids was analysed in comparison to parental genomes of each cross. In all cases the methylation pattern of the hybrid individuals resembled that of their parents: CHG and CHH methylation in the *Lyc*EPRV coding regions as well as in the IGRs could be observed (Fig.4). The unchanged methylation pattern and the absence of any virus-induced disease symptoms in the interspecific hybrids suggest that active virus was not produced by endogenous virus sequences under the conditions tested.

**Figure 5:**
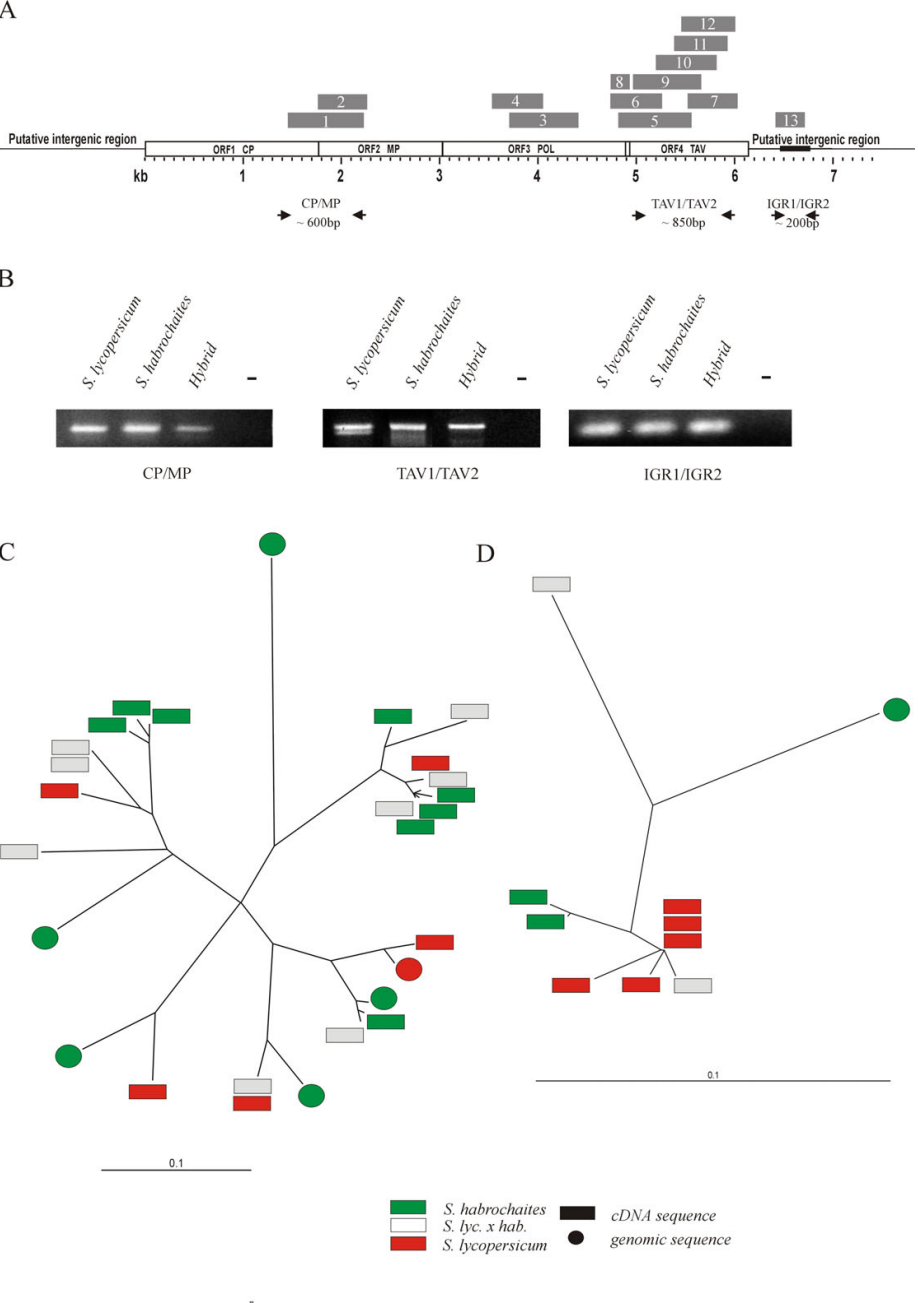
**Identification of transcripts homologous to *Lycopersicon *EPRVs**. **A. **Survey over a selection of homologous ESTs of the genus *Solanum *subsection *Lycopersicon *and their position (grey boxes) in relation to the *Lyc*EPRV structure. Details about the ESTs (according to the numbers) are given in Table 3. Arrows mark the position of primers used for RT-PCR. **B. **PolyA^+^-enriched RNA of *S.lycopersicum, S.habrochaites *and an interspecific hybrid was used for RT-PCR with primer pairs of the CP/MP and TAV ORFs and the IGR as indicated in (A). The first strand DNA template was prepared from polyA^+ ^enriched RNA from leaves of *S.lycopersicum, S.habrochaites *and an interspecific hybrid (lane 1–3 each). To detect possible genomic DNA contaminations an actin sequence spanning an intron was amplified in parallel. Water controls are indicated by a dash (lane 4 each). **C, D. **Unrooted dendrograms showing the genetic distance between genomic and cDNA sequences of the TAV region (C) and the IGR (D) of *S.lycopersicum *(red boxes), *S.habrochaites *(green boxes) and an interspecific hybrid (white boxes). cDNA sequences are indicated by a square, circles mark genomic sequences. The horizontal bar represents percent divergence (/100).

Interestingly, despite the inability to induce active virus in hybrids and the presence of cytosine methylation *Lyc*EPRVs appeared to be transcribed to some extent in healthy plants. The NCBI EST sequence databases contain transcripts from *S.lycopersicum, S.habrochaites *and *S.pennellii *with high similarity to our sequenced *Lyc*EPRVs from *S.lycopersicum *and *S.habrochaites*. More than 30 EST homologies were distributed over all four EPRV ORFs and the intergenic region. The cDNAs were derived from different tissues including flowers, red or green fruits, seeds, trichomes and shoot meristems as well as from suspension culture, callus tissue or crown galls (Fig.5A, Table 3). This suggests widespread transcription of sequences closely related to *Lyc*EPRVs in healthy tomato plants and related wild *Solanum *species not only under stress but also under normal growing conditions.

**Table 3:** Selection of ESTs from the genus *Solanum *subsection lycopersicon with homology to cloned *Lyc*EPRV sequences as shown in Fig.5A.

**No.**	**EST**	**species**	**tissue**
1	473899	*S.lycopersicum*	shoot, meristem
2	512316	*S.lycopersicum*	shoot, meristem
3	311489	*S.lycopersicum*	tomato red fruit
4	322740	*S.habrochaites*	trichome
5	247583	*S.lycopersicum*	carpel
6	511691	*S.lycopersicum*	shoot, meristem
7	465904	*S.lycopersicum*	crown gall
8	465989	*S.lycopersicum*	crown gall
9	531912	*S.lycopersicum*	callus
10	277245	*S.lycopersicum*	callus
11	414362	*S.lycopersicum*	green fruit
12	281120, 542315	*S.lycopersicum*	callus
13	245240	*S.lycopersicum*	carpel

To further study the transcriptional activity of *Lyc*EPRVs in *S.lycopersicum*, *S.habrochaites *and an inter-specific hybrid, RT-PCR was performed using the conserved primer pairs CP/MP and TAV1/TAV2 amplifying parts of the coding region and IGR1/IGR2 for the conserved box within the intergenic region (Fig.5A, B). Fragments of the expected size were amplified in all individuals (Fig.5B) and DNA sequence analysis revealed high sequence similarities to the respective *Lyc*EPRV regions. Twenty-one cDNA sequences and six genomic sequences of the TAV region comprising 761 to 806 bp each were aligned. Many turned out to be identical or nearly identical (> 98% sequence identity) on the nucleotide level whereas others diverged up to 30 to 37% (63 to 70% identity, Fig.5C). Taking into account the error-prone activity of reverse transcriptase, highly similar or identical transcripts appear to be derived from identical or corresponding EPRV copies present in both species. Nevertheless the transcripts are generally derived from more than one copy in each genome since diverging sequences are falling into at least five different clusters in *S.lycopersicum*, into four in *S.habrochaites *and six in the hybrid. None of the cloned genomic fragments of the corresponding region was matched with 100% sequence identity (97 to 99%). Many (62%) of the cDNA sequences are translationally defective, i.e. contain frameshifts and stop codons in their putative amino acid sequence. Similarly nine cDNA sequences and one genomic fragment of the IGR were analysed, which revealed higher homogeneity, but still fall into more than one cluster (Fig.5D).

### Short RNA analysis

**Figure 6:**
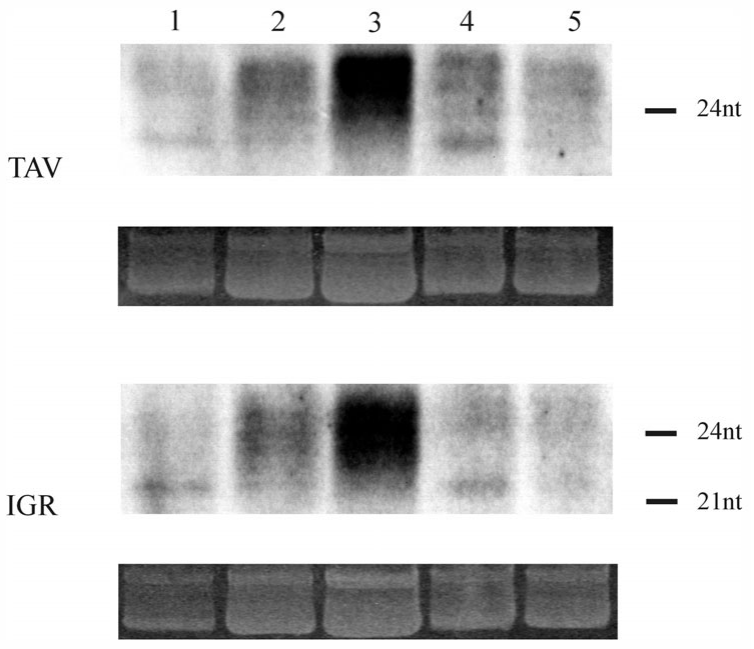
**Analysis of short RNAs homologous to *Lyc*EPRV**. The short RNA fraction of *S.lycopersicum *leaves (1, 2), *S.lycopersicum *flowers (3), *S.habrochaites *leaves (4) and leaves of an interspecific hybrid (5) was hybridized to riboprobes derived from three different TAV clones (top) and from a clone carrying the conserved part of the IGR (bottom). Ethidium bromide staining of the major RNA on the gel is shown as a loading control below each blot.

Given the absence of viral disease symptoms in plants constitutively expressing *Lyc*EPRV transcripts, we tested whether homologous short RNAs – which might be indicative of RNA-mediated silencing – were present in healthy plants. Northern blots containing short RNA fractions from leaf material of *S.lycopersicum, S.habrochaites *and an interspecies hybrid as well as flowers of *S.lycopersicum *were hybridized to RNA probes derived from the *Lyc*EPRV intergenic region and the TAV region, respectively. For the IGR probe a cDNA sequence homologous to the conserved 272 bp box served as a template. A mix of three different clones was chosen for TAV since this region is more heterogeneous. Signals could be detected in the two parental species and the hybrid with both probes and in both sense and antisense orientations. A distinct band of ~21 nucleotides in length and several bands ranging from 22–25 nucleotides in length were detected in all samples analysed. Generally, the flower-derived fraction produced the strongest signals (Fig.6).

**Figure 7:**
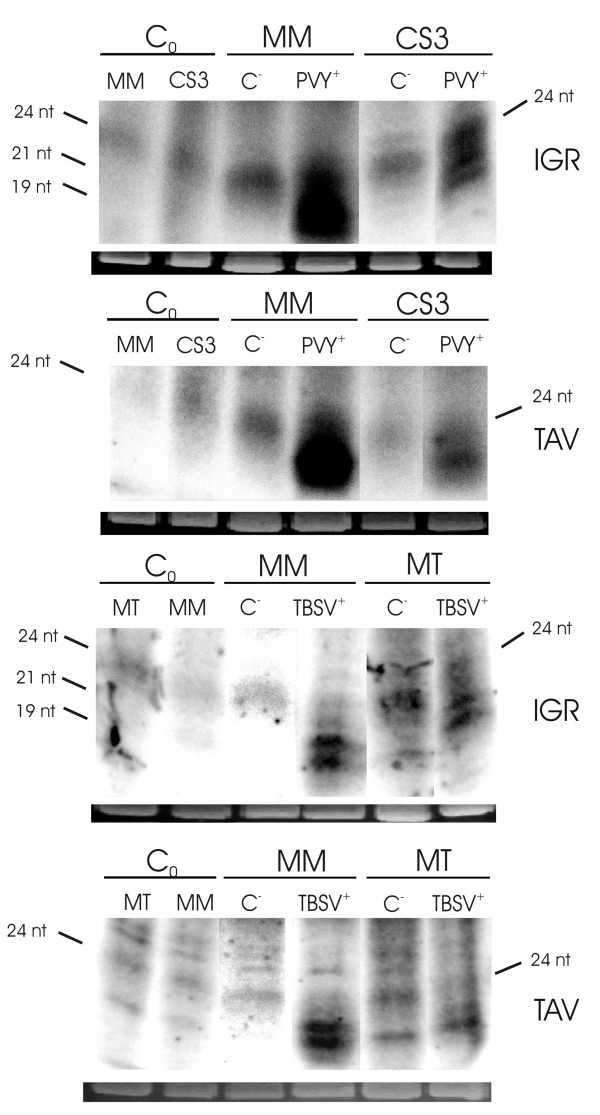
**Short *Lyc*EPRV RNAs after heterologous virus infection**. The short RNA fraction of *S.lycopersicum *leaves derived from the cultivar "Moneymaker" (MM), a transgenic line of "Moneymaker" (CS3, [63]) and the cultivar "MicroTom" (MT) was hybridized to TAV (B, D) and IGR (A, C) riboprobes after infecting the plants with PVY (*Potyvirus Y*; in A, B) or TBSV (*Tomato bushy stunt virus*; in C, D) that express suppressors of PTGS. Individual plants may show different reactions to virus infection therefore several individuals were infected in each assay. Since a general trend became visible only one representative plant is shown here. C_0_: bulked leaves harvested before infection; C^-^: mock infection; TBSV^+^: infected with *Tomato bushy stunt virus*; PVY^+^: infected with *Potyvirus Y*. Ethidium bromide staining of the major RNA on the gel is shown as a loading control below each blot.

To assess whether the short RNAs were derived from an RNAi/Post-Translational Gene Silencing (PTGS) pathway, and hence might contribute to viral defense, we analyzed short RNAs in plants infected with heterologous RNA viruses, exploiting their ability to counteract RNA silencing by encoded proteins that suppress PTGS [17,34,35]. *Potato virus Y *(PVY, *Potyvirus*) expresses HCPro, which is known to prevent the maintenance of RNA silencing and binds to siRNAs preventing the formation of the siRNA-initiated RISC assembly [39,40]. *Tomato bushy stunt virus *(TBSV, *Tombusvirus*) encodes p19, which forms homodimers and prevents the strand separation of 20–22 nt siRNA duplexes. This is a prerequisite for their integration into the RNA induced silencing complex (RISC; [[36,37], rev. in [38]]. Plants infected with either PVY or TBSV revealed increased amounts of the 21–22nt *Lyc*EPRV short RNA fraction compared to mock infected individuals and plants harvested before starting the infection procedure (Fig.7). The accumulation of the smaller sized short RNAs homologous to both the intergenic region of *Lyc*EPRVs (IGR) and part of the coding region (TAV) could be observed in the cultivars "MicroTom" as well as in "Moneymaker". The phenomenon is consistent with a formation of the *Lyc*EPRV short RNAs in the RNAi/PTGS pathway.

## Discussion

In this study, we have characterized members of a new endogenous pararetrovirus family, *Lyc*EPRV, from cultivated tomato (*Solanum lycopersicum*) and a wild relative (*S.habrochaites*). Sequence homologies in cloned fragments of genomic *Lyc*EPRV from both species lead us to conclude that they are probably derived from the same pararetrovirus. A corresponding exogenous counterpart of *Lyc*EPRV has not yet been detected, probably because the virus has not been found yet, is extinct, or has not been identified as the virus sequence could be diverged due to faster evolution of an episomal form. Hence we could also postulate TVCV as a possible origin. As shown by the DNA blot hybridization patterns (Fig.1), *S.lycopersicum *and *S.habrochaites *share similarities in *Lyc*EPRV sequence organization, but each species also has unique restriction fragments, indicating independent insertions or excisions after species separation or flanking sequence divergence. Junctions that could be amplified by PCR from *S.habrochaites *but not from *S.lycopersicum *support such species-specific insertions. The other two wild relatives tested, *S.cheesmaniae *and *S.pimpinellifolium*, have hybridization patterns strongly resembling the pattern found in *S.lycopersicum*, indicating they harbor the same organisation of *Lyc*EPRVs, although this has not been confirmed by sequence analysis. Another wild relative, *S.peruvianum *also has sequences related to *Lyc*EPRVs, but with hybridization patterns distinct from the other species investigated. The patterns of EPRV hybridization bands reflect the morphology-based taxonomy with *S.lycopersicum*, *S.pimpinellifolium *and *S.cheesmaniae *most closely related and *S.habrochaites *and *S.peruvianum *as more distant relatives [41]. Our results demonstrate that *Lyc*EPRVs and related sequences are common in many species of *Solanum *subsection lycopersicon. Given that tomato and potato are in the same genus, *Lyc*EPRVs are more similar to known EPRVs from *Nicotiana *than to the SoTu EPRV family [4] from potato.

All *Lyc*EPRV clones differed in sequence and revealed junctions between *Lyc*EPRV sequences and non-viral plant genomic sequences, indicating that the cloned sequences are indeed derived from endogenous EPRV copies in the nuclear genome, rather than from extra-genomic viral DNA. Alignment of the 14 clones of different nuclear EPRV fragments with overlapping homologous domains allowed reconstruction of a hypothetical full length *Lyc*EPRV sequence (Fig.2A) that contains all the components of a typical pararetrovirus with a structure intermediate to that of *Caulimoviruses *and *Badnaviruses *[10,42]. The coding region includes four ORFs and resembles the structure of TVCV (*Tobacco vein clearing virus*) and CsVMV (*Cassava vein mosaic virus*), two members of *Cavemoviruses*, but differs from *Caulimoviruses *with six ORFs [2,43]. This structure was confirmed in a complete coding region sequenced from BAC AC171732 recently. EPRVs from tomato, potato and tobacco share structural features including putative signals for transcription initiation and termination, and significant sequence homology, of both DNA and hypothetical proteins in the ORFs. By contrast, another endogenous pararetrovirus sequence, ePVCV from *Petunia *(also Solanaceae), differs in sequence and genomic structure [8,44].

Sequences complementary to tRNA^Met ^as a priming site for the minus-DNA strand synthesis in the intergenic region (IGR) were detected in at least a subset of copies and could be expected, since the replication of pararetroviruses is driven by transcription via RNA polymerase II and reverse transcription. Parts of the IGR with high conservation are notable, such as the 272 to 282 bp box (Fig.2B) found in all published EPRVs from tobacco, tomato and potato. Though lacking the direct repeats reported for *Ns*EPRV [1], the 272 to 282 bp conserved box of *Lyc*EPRV makes up part of the B1 box that has proved functionality as a promoter-enhancer element for *Ns*EPRV driving GUS expression in apical meristems of *A. thaliana *[15] which suggests a function, possibly as a regulatory element.

Individual *Lyc*EPRV sequences showed substantial divergence (e.g. with as little as 75% homology in the second ORF and less conservation in the intergenic region) but no sequence motifs specific to either *S.lycopersicum *or *S.habrochaites *were evident in the clones examined. Consistent with the related but distinct hybridization patterns on DNA blots, *Lyc*EPRV sequences in *S.lycopersicum *and *S.habrochaites *have a similar, although not identical, dispersed chromosomal distribution with sites scattered in pericentromeric and some intercalary heterochromatic regions, while being largely excluded from euchromatin and the NOR region (Fig.3). Individual chromosomes of both species showed characteristic stronger or weaker hybridization indicating that sequence amplification or degeneration has occurred at specific integration sites.

All EPRV containing λ-clones revealed sequence truncations and rearrangements when compared to the TVCV-like consensus structure (Fig.2A). Inverted, duplicated and truncated EPRV fragments adjacent to plant genomic DNA without viral homology have been reported for *Ns*EPRV in tobacco [1], rice EPRVs [9] and endogenous *Banana streak virus *(BSOEV) copies in banana [6]. Homologous recombination between new viral integrants, pre-existing EPRVs and perhaps retroelements could be responsible for the variable and complex genomic structures [7,10].

Half of the *Lyc*EPRV elements isolated are flanked on one or both sides by retrotransposon sequences (Table 1). Some 60% are represented by LTR sequences characteristic of the *Metaviridae *(*Ty3-gypsy*-like) elements, PCRT1 and 2 that are dispersed throughout the centromere region [30] and evidenced by the interspersed FISH signal with the LTR-homologous probe U30 (Fig.3C). In *S.habrochaites*, two of the nine *Lyc*EPRV loci were actually flanked on both sides by PCRT1. Also the tomato BAC clone AC171732 revealed PCRT1 sequences in the region flanking the *Lyc*EPRV copy. Associations of tobacco, petunia and banana EPRV sequences with *Metaviridae *elements have also been noted [1,3,6,8]. These associations may be random, due to preferential integration of either element in the other, or due to co-amplification of both elements. If retroelements constitute some 50% of the genome [24,45,46], then the association is little different from random, particularly if there is a preference for EPRVs and metaviridae elements to cluster in the genomic regions such as the centromere (see Fig.3 and [4,8] for petunia and potato). Nevertheless, it is tempting to suggest functional associations: pararetroviruses do not encode an integrase, so intact retrotransposons may supply this function in trans and related structural sites [14]. Pararetroviruses that insert into retrotransposon structures may be coamplified as chimerical structures or by template switches of RT to viral transcripts [47], in addition to other mechanisms of repetitive sequence amplification (see [48]), such as unequal and illegitimate crossing over or replication slippage of conserved short repeats as are found within the L*yc*EPRVs and related sequences.

Cytosine methylation within *Lyc*EPRV sequences was observed in both CHG and asymmetrical CHH contexts (Fig.4). CHH methylation in particular is a hallmark of RNA-directed DNA methylation in plants [19]. Similar patterns of EPRV methylation have been observed in *Petunia *[49] and *N. tabacum *[15]. There is evidence that cytosine methylation subdues EPRVs in different species. In petunia, endogenous *Petunia vein clearing virus *looses methylation upon reactivation in *Petunia *hybrida [8]. In tobacco, regulatory IGR sequences of *Ns*EPRV introduced stably into tobacco became a target of methylation and were transcriptionally silenced [15]. In rice, the copy number of endogenous *Rice tungro bacilliform virus *in different strains was directly proportional to the degree of DNA methylation and virus resistance [9]. Whether the observed cytosine methylation is responsible for transcriptionally silencing copies of *Lyc*EPRV is not known. Clearly, at least some copies of *Lyc*EPRV are transcribed, as demonstrated by the detection of transcripts derived from the *Lyc*EPRV sequences in healthy plants and homologous ESTs in databases (Fig.5). Whether these transcripts are initiated from a promoter within an EPRV sequence or from a flanking plant promoter is not known. Most ESTs correspond to the TAV region and sequence heterogeneity, including frameshifts and stop codons, suggests that the transcripts are probably non-functional and derived from more than one locus in the genome. The absence of copies with a full-length coding sequence or a functional promoter region in the genomic library does not exclude the existence of a full copy elsewhere in the genome since the cDNAs were not identical to the genomic copies sequenced. EPRV-like EST matches from normal and stressed tissue respectively were also reported for the EPRV family SoTu in the potato genome [4].

Activation of EPRVs to form virus particles that produce symptoms of infection has been reported for endogenous BSV in banana [6,11,13], endogenous TVCV in *Nicotiana edwardsonii *[2], and ePVCV in Petunia [8]. In most cases, activation occurred in interspecific hybrids and was enhanced by an additional abiotic stress (such as *in vitro *propagation/tissue culture, changes in the light regime, or frequent wounding) [2,8,11,12]. By contrast, symptoms of virus infection due to activation of latent *Lyc*EPRV were not observed in new interspecific hybrids grown under greenhouse conditions and stressed by frequent trimming. Whether this is due to stable silencing of as-yet-unidentified non-defective copies of *Lyc*EPRV in hybrids or to the general lack of potentially reactivatable copies is not known. Additionally also the absence of an asymmetric ratio of EPRV copies between parental genomes may have prevented a reactivation as this seems to enable the formation of episomal virus from integrated copies in other hybrid genomes [10,2,11]. The function of the *Lyc*EPRV transcripts in asymptomatic plants is unclear but it is tempting to speculate that they repress the pathogenicity of endogenous pararetroviruses, perhaps by an RNA-based gene silencing mechanism(s) [15]. This idea is supported by the detection of at least some CHH methylation in *Lyc*EPRVs and the presence of short RNAs with homology to *Lyc*EPRVs in healthy plants (Fig.6). Moreover the increased level of 21–22nt short RNAs in plants infected with a heterologous virus encoding suppressors of PTGS suggests a role in a constitutive RNAi/PTGS pathway. By contrast, significant amounts of short RNAs could be detected in petunia only in symptomatic tissue after activation of endogenous PVCV sequence(s) or after infection with PVCV by inoculation [49].

The presence of two size classes of short RNA, which have been implicated previously in triggering either PTGS (21 nt) or Translational Gene Silencing (TGS) and RNA-directed chromatin modifications (24 nt) [50], could provide a multi-pronged defense against endogenous or exogenous forms of the virus. The accumulation of 21–22nt *Lyc*EPRV short RNAs after heterologous virus infection with two different points of interaction in the silencing process supports the involvement of PTGS for such a defence. Given the complex and interconnected nature of RNA-mediated silencing pathways [16,51-53], and the fitness advantage of suppressing viral infection, RNA-mediated silencing of EPRVs might involve several species of short RNAs, RNA-directed DNA methylation, and both PTGS and TGS pathways.

## Methods

### Plant material and DNA isolation

Seeds of *Solanum lycopersicum *L. (syn.* Lycopersicon esculentum *Mill.) "MicroTom" were provided by Dr. A.A. Levy, Rehovot, Israel. *S.pimpinellifolium *L. (syn. *Lycopersicon pimpinellifolium *(L.) Mill.) IPK genebank accession LYC 1835,* S.cheesmaniae *(L.Riley) Fosberg (syn.* Lycopersicon cheesmaniae *L.Riley) IPK genebank accession T 675, *S.peruvianum *L. (syn.* Lycopersicon peruvianum *(L.) Mill.) IPK genebank accession T 353 and *S.habrochaites *S. Knapp & D.M. Spooner (syn. *Lycopersicon hirsutum *Dunal) IPK genebank accession T 436 were procured from the „Institut für Pflanzengenetik und Kulturpflanzenforschung“ (IPK) in Gatersleben, Germany. *S.lycopersicum *("Moneymaker") lines were obtained from D. Scharf, Frankfurt University. Plants were grown in the greenhouse. Genomic DNA was isolated from leaves with the DNeasy Plant Maxi kit (Qiagen) following the manufacturer's instructions.

### λ-library and sequencing

Two genomic DNA libraries were prepared from *Solanum lycopersicum *("MicroTom") and *S.habrochaites *using the λ-FIX II system (Stratagene) according to the protocols provided by the supplier. The libraries were screened with a subcloned 5.5 kb *Not*I-*Hin*dIII fragment of NsEPRV clone V6 corresponding to the approximate NsEPRV nucleotide positions 2–7.5 kb [1]. Λ-DNA was isolated using the Lambda Midi Kit (Qiagen) and sequenced with fluorescent chain terminators (ABI PRISM 3100 system). For analysis of DNA sequences the software programs BLAST [54] and CLUSTAL [55,56] were used, homology searches employed public domain sequence databases (GenBank, EMBL, DDBJ, SwissProt, PDB, PIR, PRF). GenBank/EMBL/DDBJ accession numbers for sequences reported in this paper are DQ273220–DQ273264.

### Southern hybridization

For Southern hybridization 1 to 2 μg of genomic DNA was sequentially digested with *Xba*I and an additional enzyme of the appropriate isoschizomer pair, fractionated on 1.5% agarose gels and transferred onto nylon membranes (Hybond N, Amersham) using standard techniques. Fragments amplified from clone Le1 with primers Le1-L: 5'GGAGGTATGACCA CGGATATAA 3'/Le1-R: 5'CCTGGTGCTAACTCTATTCCTG 3' (probe E1) and from clone Lh7 with primers Lh7-L: 5'GCAAGATATATCAGAAAGATTCC 3'/Lh7-R: 5'CCTTAGGATGGCATAGTCTG 3' (probe H7), respectively, were radiolabelled with α-[P^32^]dATP (Amersham) by random priming and hybridized onto Southern blots at 65°C in 6 × SSC overnight and washed at 65°C in 1 × SSC (saline sodium citrate)/1%(w/v) SDS (sodium dodecyl sulphate).

### RT-PCR and cDNA cloning

Total RNA was isolated from leaf material using the RNeasy Plant Mini kit (Qiagen) and enriched for polyA^+ ^RNA using the Oligotex mRNA Mini kit (Qiagen). First strand DNA was produced by Revert Aid H Minus M-MuLV Reverse Transcriptase (Fermentas) according to standard protocols and used in PCR reactions with the following primer pairs: CP: 5'CWTGTTAYAAYTGYGGAAARWTAGGAC 3'/MP: 5'TTTCWATRGGNGTATCT ATTCCTTCTC 3' and TAV1: 5'RMWDNTANHAGTCAGCAGCATGAC 3'/TAV2: 5' CATHRHYTGATCTCKTDHATARTA 3' for the coding region (annealing temperature: 50°C) and IGR1: 5'CWYTTAAGWTYATGAGTAGCTAWATTAATTTATTCCTG 3'/IGR2: 5' CCTCAAMTYTGTTTAMTCCCCTAAACGG 3' (annealing temperature 56°C) for the intergenic region (Fig.5B). An actin sequence spanning an intron was amplified in parallel to detect genomic DNA contaminations using the primer pair ActL: 5'GTTGCTATTCAGGCTGTGCT 3'/ActR: 5'TCTTTTCAATGGAGGA GCTG 3' (annealing temperature: 50°C). Reactions (50 μl) contained 50 pmol of each primer, 1.5 mM MgCl_2_, 150 μM dNTPs, 0.25U *Taq *Polymerase and ~50ng 1st strand DNA. PCR products were gel purified and cloned into the pGemT vector (Promega). In order to discriminate between different copies, cloned fragments were *Hin*fI restricted and separated on agarose gels. Fragments producing different restriction patterns were sequenced.

### Short RNA extraction and hybridization

RNA enriched for the low-molecular weight fraction (10 to 100nt) was isolated from leaves and flowers, samples of 50 μg per lane were separated on a 15% polyacrylamide gel containing 7 M urea and transferred onto nylon membranes (Hybond N^+^, Amersham) following the protocols described in [57]. The blots were hybridized with RNA probes of both orientations derived from the cloned cDNA fragments of IGRcLe-8 (DQ273223) for the intergenic region and from pooled TAVcLe-4, TAVcLe-8, TAVcLe-19 (DQ273225, DQ273229, DQ273228) for the TAV region. Hybridization conditions and probe preparation were following [57], omitting the probe fragmentation step.

### Heterologous virus infection

For mechanical transmission trials, plants at the six leaf stage were inoculated with leaf extracts from *S.lycopersicum *infected plants with *Potato virus Y *(PVY) strain PVY-NTN [58] or with *Tomato bushy stunt virus *(TBSV) strain TBSV-type [59], respectively. The virus strains were obtained from the Department of Plant Protection Virology, University of Bari, Italy. Infected leaves were ground in 0.1 M phosphate buffer (pH 7.2) with 0.2% DIECA and the extract was rubbed on celite-dusted plants. The virus spread to younger leaves after 4–6 weeks post inoculation was verified by ELISA using TBSV and PVY detection kits (LOEWE, Germany). An ELISA sample was taken as positive when its OD value was at least three times higher than the negative control values. All determinations were run in duplicate.

### Fluorescent *in situ *hybridization (FISH)

Root tips from seedlings or plants growing in pots were treated with 0.02 M 8- hydroxyquinoline, fixed in ethanol: glacial acetic acid (3:1), digested with proteolytic enzymes, and dissected in 60% (v/v) acetic acid. Chromosome preparations were either made by squashing [60] or spreading [61]. Flower buds were fixed untreated and anthers were dissected and the stage of meiosis determined to be pachytene, before they were processed as above.

The ribosomal probe (clone pTa71), contains a 9 kb *Eco*RI fragment of the repeat unit of 25S-5.8S-18S rDNA from *T. aestivum *[62]. Part of the dispersed middle repetitive tomato sequence U30 [31] was amplified and cloned from *S.lycopersicum *(DQ273250). Mixtures of four probes each for *S.lycopersicum *and *S.habrochaites *(*Lyc*EPRV-Sl, *Lyc*EPRV-Sh) were selected (Table 2). PCR amplified inserts of clones were labelled with biotin 16-dUTP (Roche) or digoxigenin 11-dUTP (Roche) by random priming (Bioprime & Random primer kit; Invitrogen).

*In situ *hybridization followed [60]. The hybridization mixture consisted of 50 to 100 ng/slide of each probe, 50% (v/v) formamide, 2 × SSC, 10% (v/v) dextran sulphate, 0.12% (w/v) SDS, 0.12 mM EDTA (ethylene-diamine-tetra-acetic acid) and 1 μg/μl salmon sperm DNA. After overnight hybridization, slides were washed in 20% (v/v) formamide/0.1 × SSC at 42°C, giving a hybridization stringency of 85%. Hybridization sites were detected by streptavidin conjugated to Alexa 594 (Molecular Probes) or FITC (fluorescein isothiocyanate) conjugated anti-digoxigenin antibody (Roche) in 4 × SSC, 0.1% (v/v) Tween-20, 5% (w/v) BSA (bovine serum albumin). Preparations were stained with DAPI (4'-6-diamidino-2-phenylindole) and analysed on an Axioplan 2 epifluorescence microscope (Zeiss) with single band pass filters equipped with a cooled colour CCD camera (Optronics, model S97790). FISH and DAPI images were overlaid using the RGB channels of Adobe Photoshop CS and CS2 software; DAPI images were sharpened using the Gaussian deblur function and colour balance and processing of the FISH signal was achieved using only those function that treat all pixels equally. For the pachytene overlay figures (Figs. 3E and 3F) the captured colour images were converted to gray image, enhanced and overlaid: DAPI images were left B&W and the FISH signals were falsely coloured red and green, respecively. Each hybridization experiment was at least carried out twice and for each probe eight to twenty cells were analysed.

## Authors' contributions

CS carried out sequencing and bioinformatics, methylation and expression analysis as well as the detection of short RNAs, participated in and helped designing the FISH experiments and virus infections and drafted the manuscript. WG and MFM made the λ-libraries and the interspecific crosses. CHT carried out some FISH experiments, EGBF and MLCM carried out the heterologous virus infection, MM initiated and coordinated the study and helped drafting the manuscript, TS supported the coordination of the study, especially the design and analysis of the FISH experiments, and the drafting of the manuscript. All authors read and approved the final manuscript.

## Supplementary Material

Additional file 1 **Prometaphase (A) and pachytene (B) chromosomes of *S.lycopersicum *"MicroTom" after fluorescent in situ hybridization with LycEPRV-Sl (red) and U30 repetitive sequence (green)**. The U30 signal covers most of the pericentromeric heterochromatin stained strongly with DAPI (blue) while LycEPRV-Sl has fewer hybridization sites. Bar equals 10 μm. Click here for file
