# Supplementary figures and images for: Endogenous pararetroviral sequences in tomato (Solanum lycopersicum) and related species
Source: BMC Plant Biol. 2007 May 21;7:24. doi: 10.1186/1471-2229-7-24 (PMC1899175; doi:10.1186/1471-2229-7-24)

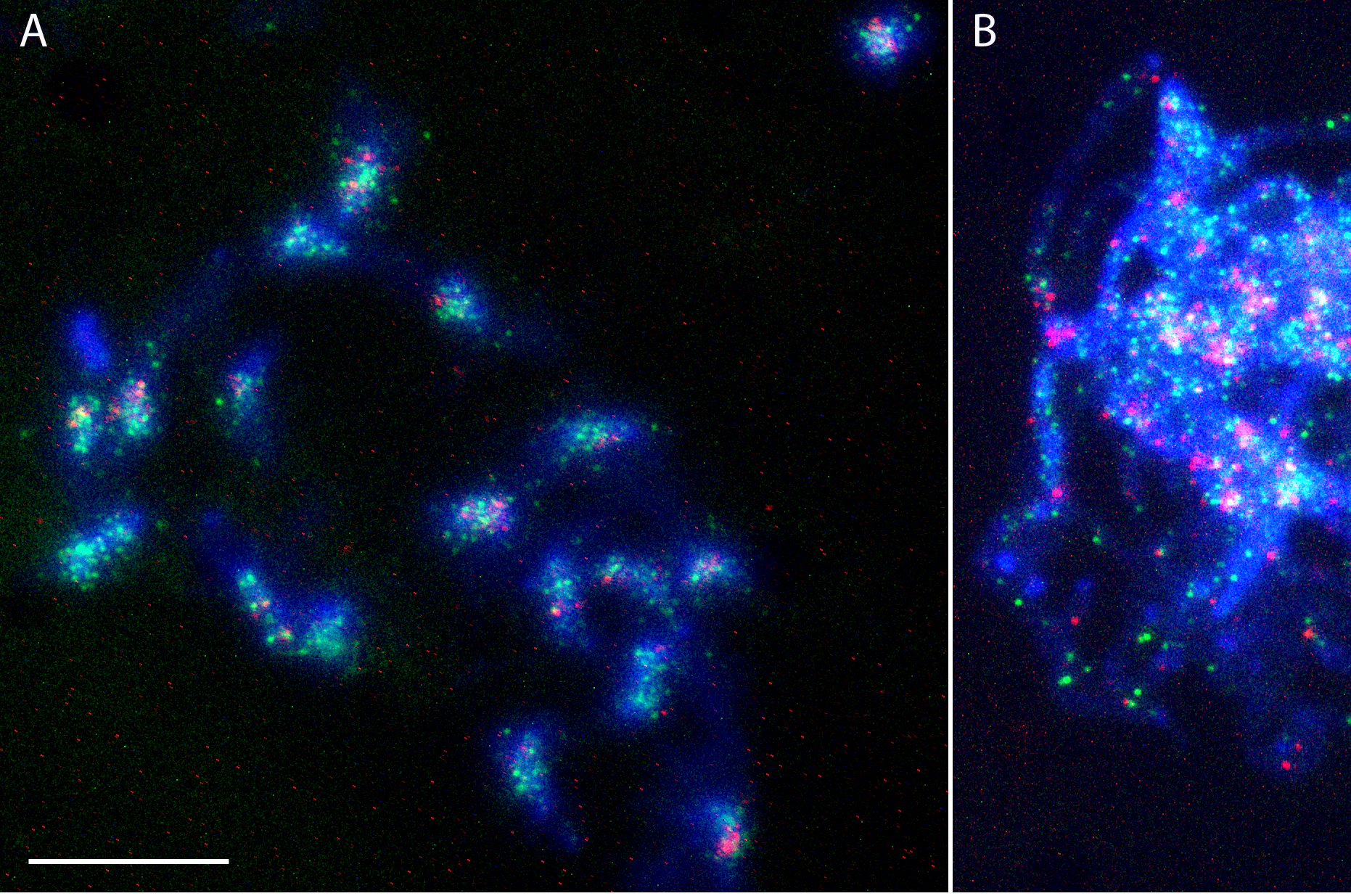

Supplement: Additional file 1 — Prometaphase (A) and pachytene (B) chromosomes of S.lycopersicum "MicroTom" after fluorescent in situ hybridization with LycEPRV-Sl (red) and U30 repetitive sequence (green). The U30 signal covers most of the pericentromeric heterochromatin stained strongly with DAPI (blue) while LycEPRV-Sl has fewer hybridization sites. Bar equals 10 μm. [file 1471-2229-7-24-S1.jpeg]
